# Modelling of Running Performances: Comparisons of Power-Law, Hyperbolic, Logarithmic, and Exponential Models in Elite Endurance Runners

**DOI:** 10.1155/2018/8203062

**Published:** 2018-10-03

**Authors:** H. Vandewalle

**Affiliations:** UFR de Santé, Médecine et Biologie Humaine, Université Paris XIII, Bobigny, France

## Abstract

Many empirical and descriptive models have been proposed since the beginning of the 20^th^ century. In the present study, the power-law (Kennelly) and logarithmic (Péronnet-Thibault) models were compared with asymptotic models such as 2-parameter hyperbolic models (Hill and Scherrer), 3-parameter hyperbolic model (Morton), and exponential model (Hopkins). These empirical models were compared from the performance of 6 elite endurance runners (P. Nurmi, E. Zatopek, J. Väätäinen, L. Virén, S. Aouita, and H. Gebrselassie) who were world-record holders and/or Olympic winners and/or world or European champions. These elite runners were chosen because they participated several times in international competitions over a large range of distances (1500, 3000, 5000, and 10000 m) and three also participated in a marathon. The parameters of these models were compared and correlated. The less accurate models were the asymptotic 2-parameter hyperbolic models but the most accurate model was the asymptotic 3-parameter hyperbolic model proposed by Morton. The predictions of long-distance performances (maximal running speeds for 30 and 60 min and marathon) by extrapolation of the logarithmic and power-law models were more accurate than the predictions by extrapolation in all the asymptotic models. The overestimations of these long-distance performances by Morton's model were less important than the overestimations by the other asymptotic models.

## 1. Introduction

Many models [1–11] of running performances based on biomechanics and physiology have been proposed. These models are generally complex. For example, the physiological model proposed by Péronnet and Thibault [7] included the inertia, power, and capacity of the anaerobic and aerobic metabolisms.

Empirical and descriptive models have also been proposed since the beginning of the 20^th^ century and presented in many reviews [12–21]. Empirical models are derived by observation and experimentation rather than by theoretical considerations [14]. The empirical models are less complex than the biomechanical and physiological models but are also less explicative. The most famous empirical models corresponded to a power-law model (Kennelly, 1906), asymptotic hyperbolic models (Hill, 1927; Scherrer, 1954), and, more recently, a logarithmic model (Péronnet and Thibault, 1987) and 3-parameter asymptotic models (Hopkins, 1989; Morton, 1996). The asymptotic models correspond to horizontal asymptote equations: the functions approach a horizontal line when t_lim_ tends to infinity. In these models, it is assumed that the speeds lower than these asymptotes can be maintained infinitely.

The empirical models of running exercises are often used to estimate

(i) the improvement in performance [22]
(ii) the effects of age [23, 24] and sex [25, 26] on running performance
(iii) the future performances and running speeds over given distances
(iv) the endurance capability [7, 8], that is, “the ability to sustain a high fractional utilization of maximal oxygen uptake for a prolonged period of time”
(v) the speed of training sessions [27]
(vi) the maximal aerobic speed [7, 8]

The maximal aerobic speed, otherwise known as MAS, is the lowest running speed at which maximum oxygen uptake (V02 max) occurs, and is also referred to as the velocity at V02 max (vV02 max). MAS is useful for training prescription and monitoring training loads. Péronnet and Thibault suggested estimating MAS by computing the maximal speed corresponding to 7 min [8]. The maximal lactate steady state, defined as the highest constant power output that can be maintained without a progressive increase in blood lactate concentration, is usually sustainable for 30 to 60 min. [28–30].

The first studies on the modelling of running performances were based on the world records because these records measured under standard external conditions represent the most reliable index of human performance [31, 32]. The running times of the slower runners are more variable than those of the faster runners [33]. The best performances of world elite runners are probably very close to their maximal performances because they generally correspond to the results of many competitions against other elite runners and the motivation is probably optimal during these races. Now, the best performances of elite endurance runners who ran on different distances and were the best of their times can be found on the Internet (Wikipedia, etc.). Therefore, it is possible to study the characteristics of the different models which have been proposed for endurance exercises with the best performances of elite endurance runners.

The performances of different runners were used in each study on the modelling of world and Olympic records [7, 22, 31, 32, 34, 35]. In contrast, in the present investigation, each model was computed only from the performances of a single runner. The computations of each model were repeated for different world elite endurance runners (P. Nurmi, E. Zatopek, J. Väätäinen, L. Virén, S. Aouita, and H. Gebrselassie) who were world-record holders and/or Olympic winners and/or world or European champions. They participated several times in international competitions over the same distances (1500, 3000, 5000, and 10000 m) that corresponded to a large range of distances. Their best individual performances are presented in Table 1.

Moreover, if a model is not perfect for a large range of performances, the values of its parameters computed from different ranges of distances will be significantly different. In the present study, the parameters of the different models were computed with 3 ranges of distances:

(i) 1500-3000-5000-10000 m for the largest range
(ii) 1500-3000-5000 m, which is equivalent to the range of t_lim_ generally used in the studies on critical speed or critical power (from 3 to 15 min)
(iii) 3000-5000-10000 m, which corresponds to exercises slower than maximal aerobic speed

Several previous investigations studied the evolution of the parameters in the models of running performances at different times [22, 34]. Similarly, the six elite endurance athletes of the present study ran at different times and their performances were performed in different conditions (cinder tracks versus synthetic tracks, nutrition, etc.) and were the results of different running exercises (for example, an equivalent of* fartleck* for Nurmi, an equivalent of interval-training for Zatopek, and altitude training for Gebrselassié), which could partly explain the evolution of the performances in these world elite runners and could also change the best model of individual running performances.

The present study (1) applied the power-law and logarithmic models and four asymptotic models (two 2-parameter hyperbolic models, a 3-parameter hyperbolic model, and a 3-parameter exponential model) to the individual performances of the elite runners, (2) compared the accuracy of these models and the effects of the range of performances on their parameters to assess which is the best model, and (3) compared the predictions of MAS by interpolation and the prediction of maximal running speeds for long distances (30, 60 min and also marathon in 3 runners) by extrapolation.

## 2. History of the Power-Law, Hyperbolic, Logarithmic, and Exponential Models

### 2.1. Power-Law Model (Kennelly)

In 1906, Kennelly [12] studied the relationship between running speed (S) and the time of the world records (t_lim_) and proposed a power law:

(1) $$\begin{array}{c} D_{lim}=k{t_{lim}}^{g} \end{array}$$

where k is a constant and g an exponent. This power law between distance and time corresponds to a power law between time and speed (S):

(2) $$\begin{array}{c} S=\frac{D_{lim}}{t_{lim}}=\frac{k{t_{lim}}^{g}}{t_{lim}}=k{t_{lim}}^{g\text{ - }1} \end{array}$$

Exponent g is probably an expression of endurance capability. Indeed, the t_lim_-D_lim_ relationship would be perfectly linear if g is equal to 1. It is likely that the curvatures of the t_lim_-S and t_lim_-D_lim_ relationships depend on the decrease in the fraction of maximal aerobic metabolism that can be sustained during long lasting exercises. The value of exponent g is independent of scaling as it is independent of the expression of t_lim_, S, and D_lim_.

In theory, parameter k should be correlated to maximal running speed because k is equal to the maximal running speed corresponding to one second. Indeed, when t_lim_ is equal to 1s

(3) $$\begin{array}{c} S=k{t_{lim}}^{g\text{ – }1}=k*1^{g\text{ - }1}=k*1=k \end{array}$$

In 1981, a similar power-law model was proposed by Riegel [36]:

(4) tlim=aDlimb

(5) S=Dlimtlim=DlimaDlimb=Dlim1  -  ba

As D_lim_ = kt_lim_^g^

(6) Dlim1/g=ktlimg1/g=k1/gtlimtlim=Dlim1/gk1/g=Dlim1  -  baa=k1/g

and

(7) Dlim1/g=Dlim1  -  b1g=1  -  bb=1  -  1g=g  –  1g

These equations of Riegel have recently been applied to a large study on 2303 recreational endurance runners [37].

### 2.2. Hyperbolic Model (Hill, Scherrer)

In 1927, Hill [1] proposed a hyperbolic model to describe the world-record curve in running and swimming. Hill observed that the “running curve,” or the relationship between a runner's power output (P) and the total duration of a race (T), can be described by a hyperbolic function:

(8) $$\begin{array}{c} P=\left(\;\frac{A}{T}\;\right)+R \end{array}$$

where A and R represent the capacity of anaerobic metabolism and the rate of energy release from aerobic metabolism, respectively. In 1954, Scherrer et al. proposed a linear relationship [38] between the exhaustion time (t_lim_) of a* local exercise* (flexions or extensions of the elbow or the knee) performed at different constant power outputs (P) and the total amount of work performed at exhaustion (W_lim_) for t_lim_ ranging between 3 and 30 minutes:

(9) $$\begin{array}{c} W_{lim}=a+{bt}_{lim} \end{array}$$

Consequently, the relationship between P and t_lim_ is hyperbolic:

(10) Wlim=Ptlim=a+btlimtlim=aP  –  b

After the publication of an article in English (1965) by Monod and Scherrer [39], Ettema (1966) applied the critical-power concept to world records in running, swimming, cycling, and skating exercises [40] and proposed a linear relationship between D_lim_ and t_lim_ for world records from 1500 to 10000 m:

(11) $$\begin{array}{c} D_{lim}=a+{bt}_{lim} \end{array}$$

where t_lim_ corresponded to the world record for a given distance (D_lim_). It was assumed that the energy cost of running, i.e., the energy expenditure per unit of distance, was almost independent of speed under 20 km.h^−1^. Consequently, D_lim_ and parameter a were equivalent to amounts of energy. Therefore, parameter a has been interpreted as equivalent to an energy store and an estimation of maximal Anaerobic Distance Capacity (ADC expressed in metres) for running exercises whereas slope b was considered as a critical velocity (S_Crit_).

(12) Dlim=ADC+SCrit1tlim

(13) tlim=ADCS  –  SCrit1

However, the linear W_lim_-t_lim_ was an approximation as indicated by Scherrer and Monod (1960): “The relationship W = f(t) is not perfectly linear as shown on Figure 2(a), where the curves tend towards abscissa beyond 30 minutes” [41]. In the study by Ettema in 1966, S_Crit_ and ADC depended on the range of t_lim_, which was confirmed by more recent studies [42, 43].

In 1981, the linear W_lim_-t_lim_ relationship was adapted to exercises on a stationary cycle ergometer and it was demonstrated that slope b of the W_lim_-t_lim_ relationship was highly correlated with the ventilatory threshold [44]. Therefore, slope b was proposed as an indicator of general endurance and the concept of critical power or critical velocity was again studied. Different equations were proposed for the estimation of S_Crit_ (or CP). For example, S_Crit_ on a treadmill [45] was computed from the linear relationship between D_lim_ and the inverse of t_lim_ (1/t_lim_):

(14) $$\begin{array}{c} S=a\left(\;\frac{1}{t_{lim}}\;\right)+b={ADC}_{2}\left(\;\frac{1}{t_{lim}}\;\right)+S_{Crit2} \end{array}$$

More recently, Morton [15] proposed a fourth model for the critical power, a nonlinear model including a third parameter corresponding to maximal instantaneous power (P_max_). This model has been adapted to running exercises with an instantaneous maximal running speed (S_Max_):

(15) $$\begin{array}{c} t_{lim}=\frac{{ADC}_{3}}{\left(S\text{ – }S_{Crit3}\right)}\;\;–\;\;\frac{{ADC}_{3}}{\left(S_{Max}\text{ - }S_{Crit3}\right)} \end{array}$$

Actually, the different asymptotic hyperbolic models are the most used and studied [46].

### 2.3. Logarithmic Model (Péronnet-Thibault)

The metabolic model proposed by Péronnet and Thibault [7, 8] included factors that took into account the contributions of aerobic and anaerobic metabolism to total energy output according to the duration of the race. The inertia of the aerobic metabolism at the beginning of the exercise was also included in the model. In addition, the use of anaerobic store S_A_ was assumed to decrease beyond T_MAP_ (exhaustion time corresponding to maximal aerobic power):

(16) SA=Afor  T≤TMAPSA=A  –  0.233Aln⁡TTMAPfor  T>TMAP

A runner is only capable of sustaining his maximal aerobic power for a finite period of time. The performances in long distance events depend on the ability to utilize a large percentage of V_O2_max over a prolonged period of time (endurance capability). Péronnet and Thibault [7, 8] assumed that t_lim_ corresponding to maximal aerobic speed (t_MAS_) is equal to 7 min. They proposed the slope (E) of the relationship between the fractional utilization of MAS and the logarithm of t_lim_/7min (420 s) as an index of endurance capability:

(17) S=MAS  –  E7minln⁡tlim420100 SMAS=100  –  Eln⁡tlim420

where MAS is the maximal running speed corresponding to 7 min and E is the endurance index corresponding to MAS (E =100 E_7min_/MAS). There was a significant correlation between the ventilatory threshold and E in marathon runners [47], which suggested that E was an index of aerobic endurance. The values of E and MAS_7min_ can be estimated from two running performances with a nomogram [48].

### 2.4. Exponential Model

Hopkins et al. [13] have presented an asymptotic exponential model for short-duration (10 s - 3 min) running exercises on a treadmill with 5 different slopes (9 to 31%). This model was

(18) $$\begin{array}{c} I_{t}=I_{\infty }+\left(\;I_{0}\;\;–\;\;I_{\infty }\;\right)\exp\left(\;–\frac{t_{lim}}{\tau }\;\right) \end{array}$$

where I_∞_ is the slope corresponding to infinite time, I_0_ the slope corresponding to a time equal to zero, I_t_ the slope corresponding to t_lim_, and *τ* is a time constant. This model can be adapted to running exercises on a track:

(19) $$\begin{array}{c} S=S_{\infty }+\left(\;S_{0}\text{ – }S_{\infty }\;\right)\exp\left(\;–\frac{t_{lim}}{\tau }\;\right) \end{array}$$

This asymptotic exponential model derived from Hopkins' model has been used and compared to the different asymptotic hyperbolic models in several studies [49–52].

## 3. Methods

The logarithmic, power-law, and hyperbolic models which are 2-parameter models were computed by linear least-square regressions between time data and speed data (or distance data). Time data correspond to t_lim_ or the logarithm of t_lim_. Speed data correspond to speed or the logarithm of speed. The models by Morton and Hopkins are 3-parameter models whose individual regressions were computed by an iterative least square method.

### 3.1. Computation of the Empirical Models

#### 3.1.1. Computation of the Power-Law Model

If Y = A∗X, the logarithm of Y is equal to

(20) $$\begin{array}{c} \ln\left(\;Y\;\right)=\ln\left(\;A\;\right)+\ln\left(\;X\;\right) \end{array}$$

If Y = X^-B^, the logarithm of Y is equal to

(21) $$\begin{array}{c} \ln\left(\;Y\;\right)=\text{-}B\ln\left(\;X\;\right) \end{array}$$

If Y = A∗X^-  B^, the logarithm of Y is equal to

(22) $$\begin{array}{c} \ln\left(\;Y\;\right)=\ln\left(\;A\;\right)\text{- }B\ln\left(X\right)=C\text{ - }B\ln\left(\;X\;\right) \end{array}$$

where C = ln(A) and exp(C) = exp[ln(A)] = A.

Therefore, the power laws between t_lim_ and D_lim_ or S can be determined by computing the regression between the natural logarithms of D_lim_ and t_lim_:

(23) lnDlim=α+γln⁡tlim=ln⁡k+gln⁡tlimk=eln⁡k=eα

#### 3.1.2. Computation of the Hyperbolic Models

In the present study, three estimations of critical velocity (S_Crit1_, S_Crit2_, and S_Crit3_) were computed:

12  bisDlim=ADC1+SCrit1tlimY=α1+β1X

where Y = D_lim_; X = t_lim_; *α*_1_ = ADC_1_; *β*_1_ = S_Crit1_

14  bisS=a+b1tlim=SCrit2+ADC21tlimY=α2+β2X

where Y = S; X = 1/t_lim_; *α*_2_ = S_Crit2_; *β*_2_ = ADC_2_

In the 3-parameter model by Morton

(15) $$\begin{array}{c} t_{lim}=\frac{{ADC}_{3}}{\left(S\text{ – }S_{Crit3}\right)}\;\;–\;\;\frac{{ADC}_{3}}{\left(S_{Max}\text{ - }S_{Crit3}\right)} \end{array}$$

Let C = ADC_3_/(S_Max_ - S_Crit3_)

(24) tlim=ADC3S  –  SCrit3  –  CSMax=SCrit3+ADC3C

First, this equation was computed by an iterative least square method for a hyperbolic decay formula with 3 parameters (Y_0_, a, and b):

(25) $$\begin{array}{c} Y=Y_{0}+\frac{ab}{\left(x+b\right)} \end{array}$$

where Y_0_ = - C, b = - S_Crit3_, and ab = ADC_3_

Unfortunately, there was no convergence of the iteration. Therefore, an iteration was tested for another equation:

24  bistlim+C=ADC3S  –  SCrit3S  –  SCrit3=ADC3tlim+CS=SCrit3+ADC3tlim+C

This equation was computed with an iterative least square method for a similar hyperbolic decay formula with 3 parameters (Y_0_, a, and b):

(26) $$\begin{array}{c} Y=Y_{0}+\frac{ab}{\left(x+b\right)} \end{array}$$

where Y = S, Y_0_ = S_Crit3_, ab = ADC_3_, and b = C.

As the value of S_max_ = S_Crit3_ + ADC/C

(27) $$\begin{array}{c} S_{max}=Y_{0}+\frac{ab}{b}=Y_{0}+a \end{array}$$

Fortunately, there was a convergence in the iteration for this equation.

#### 3.1.3. Computation of the Logarithmic Model

The value of E was estimated by computing the regression between S and the logarithm of t_lim_/420 for the different distances:

(28) $$\begin{array}{c} S=\alpha \text{ – }\beta \ln\left(\;\frac{t_{lim}}{420}\;\right) \end{array}$$

When t_lim_ = 420, S is equal to MAS and ln(t_lim_/420) is equal to 0. Therefore

(29) S=MAS=α+0E=100βMAS=100βα

#### 3.1.4. Computation of the Exponential Model

At least three distances are necessary to compute Hopkins' model (see (19)) which is a three-parameter model (S_*∞*_, a_1_, and b_1_) like Morton's model.

(30) S=S∞+S0  –  S∞exp⁡−tlimτ=S∞+aexp⁡–btlim

The regressions were computed by an iterative least square method for a single exponential decay formula with 3 parameters (Y_0_, a, and b):

(31) $$\begin{array}{c} Y=Y_{0}+\alpha \exp\left(\;-\beta X\;\right) \end{array}$$

where X = t_lim_, Y_0_ = S_∞_, *α* = a, and *β* = b

### 3.2. Estimations of Maximal Running Speeds corresponding to 7, 30, and 60 Minutes

The estimations of the individual maximal running speeds corresponding to 7 minutes (estimation of maximal aerobic speed, MAS) were performed by interpolation from the 1500-3000-5000m performances.

The estimations of the maximal running speed during 30 min were done by extrapolation from the 1500-3000-5000m performances. The 30-min running times were compared with the 10000 m performances (S_10000_).

The estimations of the maximal running speed during 60 min were done by extrapolation from the 1500-3000-5000-10000 m performances.

### 3.3. Accuracy of the Estimations of Running Speed

The individual running speeds corresponding to the different distances (1500, 3000, 5000, and 10000 m) were estimated from the individual regressions of the different models and compared with the actual speeds for the same distances. First, for each model, the individual running speeds corresponding to t_lim_ between 1 and 1900 s were computed from the individual regressions with an increment equal to 1 s. Secondly, the individual relationships between distance and the estimated value of t_lim_ were computed by multiplying t_lim_ and the corresponding estimated speed (distance = speed x time). Then, the individual estimated values of running speed corresponding to 1500, 3000, 5000, and 10000 m were registered and compared with the actual values of running speeds.

Thereafter, the ratios of estimated speed to actual speed were computed for each distance and each runner.

### 3.4. Statistics

All the computations of the model and the statistics were performed with the SigmaPlot software (Systat, Chicago, USA).

#### 3.4.1. Comparisons of the Parameters

The comparisons of the parameters, computed from different ranges of distances or from different running models (S_Crit1_, S_Crit2_, S_Crit3_, S_∞_, S_Max_, and S_0_), were studied with a nonparametric paired test (Wilcoxon signed rank test) since the sample sizes were low (6 runners). Significance was accepted at critical P<0.05. The probability was equal to 0.031 in Wilcoxon signed rank test when all the individual values of a parameter are either lower or higher than all the corresponding individual values of a parameter in another model (or another performance range).

#### 3.4.2. Comparison of the Accuracy in the Different Models

In statistics, the sum of the squares of residuals (deviations predicted from actual empirical values of data) is a measure of the discrepancy between the data and an estimation model. A small sum of the squares of residuals indicates a tight fit of the model to the data.

However, in the present study, the comparisons of the accuracy in the different models cannot be based on the differences in the sums of the squares of residuals because the residuals in the power-law model corresponded to the logarithm of the residuals and because the individual regression of the first hyperbolic model (S_Crit1_) did not correspond to regressions between t_lim_ and running speeds (S) but regressions between t_lim_ and distances (D_lim_). Moreover, it would be assumed that there was a homoscedasticity in the residuals of the running speeds, which could not be tested with only 4 datasets in an individual regression. In addition, the residuals of computed running speeds could be more important in the faster runners. In the present study, the residuals were computed as equal to the differences between 1 and the ratios of estimated speed to actual speed for each distance and each runner. For a given running model, the squares of these residuals were computed for each distance and each runner, which corresponded to 24 squares (4 distances x 6 runners). The values of the squares of a model were compared with the values of squares for the same distances and same runners in another model. The statistical significance values of the 24 paired differences between two running models were tested with paired Student's* t*-tests after normality tests (Kolmogorov-Smirnov tests). When the normality tests failed, the paired Student's* t*-tests were replaced with the Wilcoxon signed rank tests.

In addition, for each runner, the sum of squared errors for the four distances was computed for each model. The square root of the mean of this sum (root mean square error, RMSE) was computed for each runner and each model. A large error has a disproportionately large effect on RMSE which is, consequently, sensitive to outliers.

## 4. Results

### 4.1. Power-Law Model Applied to Elite Runners

The effects of the distance range were not significant for exponent g (0.063 < P < 0.125) as well as parameter k (0.063 < P < 0.094).

**Figure 1:**
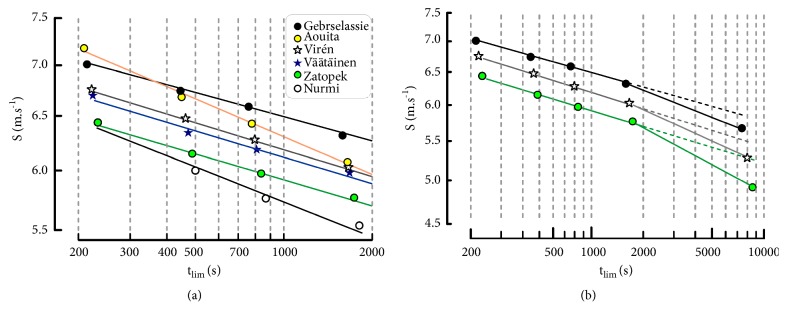
(a) Individual linear relationships (power-law model) with logarithmic scales for running speed and t_lim_. The performances by Nurmi and Zatopek were the same for the 1500 m distance. (b) Extrapolation of the linear relationships (dashed lines) to marathon performances.

The estimations of the logarithm of running speeds (S) were close to the logarithm of actual speeds (Figure 1(a)). The correlation coefficients of the individual linear relationships (see (5)) between ln(S) and ln(t_lim_) or ln(D_lim_) and ln(t_lim_) were higher than 0.999 in all the runners for 1500-10000m.

Similarly, the ratios of estimated to actual speeds (Table 3) for the four distances were accurate: the errors were lower than 1%, except the 10000 m performance by Nurmi (error equal to 1.1%).

Marathon performances were under the extrapolation of the lines of regression computed from the 1500-10000 m track performances (Figure 1(b)).

### 4.2. Hyperbolic Model Applied to Elite Endurance Runners

#### 4.2.1. S_Crit1_ Model

**Figure 2:**
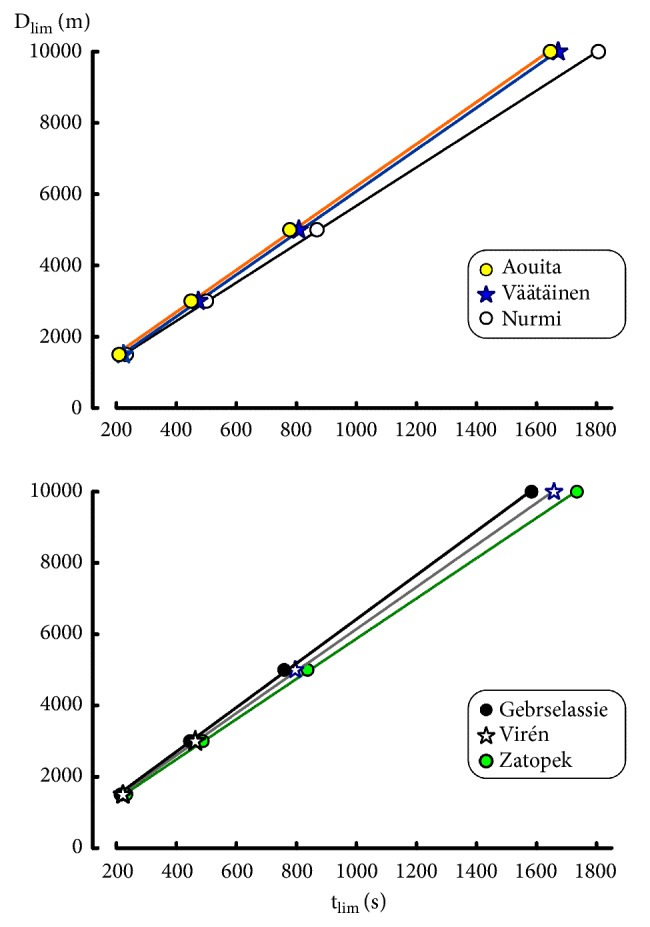
Linear relationships between exhaustion time (t_lim_) and distance (D_lim_).

The linear relationships between time (t_lim_) and distance (D_lim_) are presented in Figure 2. For all the runners, the correlation coefficients of the linear regression between t_lim_ and D_lim_ were higher than 0.999 for the different ranges of D_lim_. Parameters S_Crit1_ and ADC_1_ are presented in Table 4. As in previous studies on critical power [42, 43], the values of S_Crit1_ depended of the range of t_lim_. All the differences in S_Crit1_ and ADC_1_ were significant (P = 0.031 in the Wilcoxon signed rank test): the values S_Crit1_ computed from 1500 to 5000m were significantly higher than S_Crit1_ computed from 3000 to 10000m. The ratios of the estimated running speeds to the actual speed estimated from S_Crit1_ model are presented in Table 5. The errors are moderate (< 2%) except for 1500 m.

**Figure 3:**
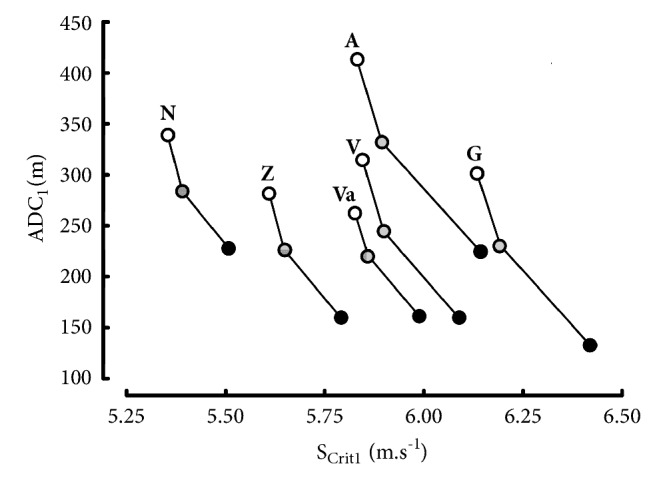
Relation between critical speed and Anaerobic Distance Capacity (ADC_1_) for different ranges of distances: 1500 to 5000 m (black dots), 3000 to 10000 m (empty circles), and 1500 to 10000 m (grey dots).

The values of ADC_1_ largely depended on the range of performances as shown in Figure 3. When the individual critical speeds decreased because of a change in the range of performances, the corresponding ADC_1_ increased. These increases in ADC_1_ were much more important than the decrease in S_Crit1_. For example, S_Crit1_ computed from 3000-10000 m was 3.8% lower than S_Crit1_ computed from 1500-5000 m (Table 3) whereas the corresponding increase in ADC_1_ was equal to 79% (319 ± 53 m versus 178 ± 39 m, Figure 3).

#### 4.2.2. S_Crit2_ Model

**Figure 4:**
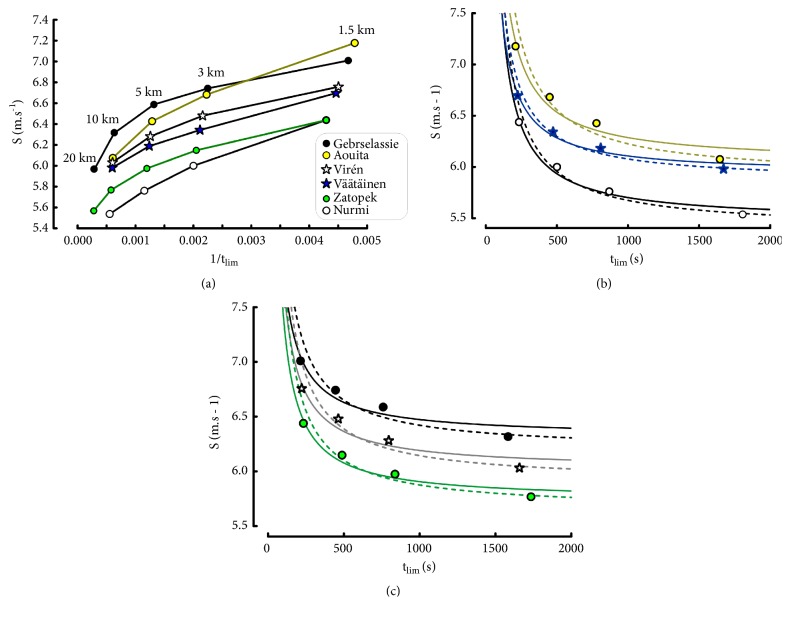
(a) Individual S-1/t_lim_ relationships in elite endurance runners. ((b) and (c)) Individual hyperbolic curves corresponding to S_Crit1_ model (dashed curves) and S_Crit2_ model (solid curves).

The individual S-1/t_lim_ relationships were not linear (Figure 4(a)) when long distances (10 km) were included. The correlation coefficients of the linear regressions between 1/t_lim_ and D_lim_ were equal to 0.976 ± 0.0126. Parameters S_Crit2_ and ADC_2_ depended on the range of distances (Table 6). All the differences in S_Crit2_ and ADC_2_ in function of the distance ranges were significant (P = 0.031). When S_Crit2_ decreased because of a change in the range of performances, the corresponding ADC_2_ increased. These variations in ADC_2_ were much more important than the variation in S_Crit2_ (Table 6).

#### 4.2.3. Comparison of the S_Crit1_ and S_Crit2_ Models

**Figure 5:**
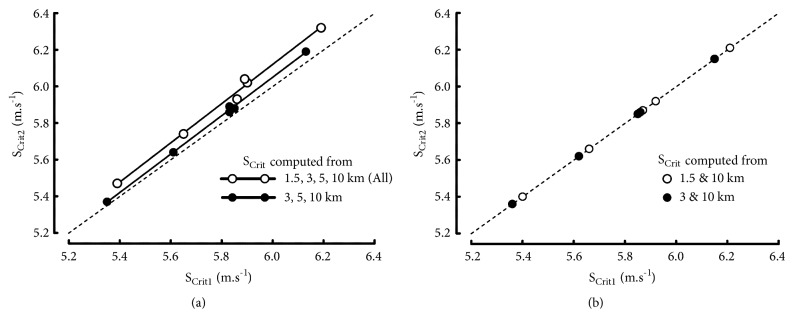
(a) Relationships between the individual values of S_Crit1_ and S_Crit2_ computed from 3 distances (black dots) or 4 distances (empty circles). (b) Relationships between S_Crit1_ and S_Crit2_ computed from 2 distances, only.

As in previous studies [49–52], the estimates of S_Crit_ differed according to the mathematical model used to describe the speed-t_lim_ relationships. The values of S_Crit2_ (Table 6) were significantly higher (P = 0.031) than S_Crit1_ (Table 4). Indeed, the values of S_Crit1_ were slightly lower in all the elite endurance runners than the value of S_Crit2_ when they were computed with three (3-5-10km) or four (1.5-3-5-10km) distances (Figure 5(a)). When short distances (1500 m) were included, the differences between S_Crit1_ and S_Crit2_ increased as demonstrated in Figure 5(a). However, S_Crit1_ and S_Crit2_ computed from the same range of performance were highly correlated (P ≥ 0.996). The values of ADC_2_ (Table 6) were significantly lower (P = 0.031) than ADC_1_ (Table 4) but were significantly correlated (0.940 < r < 0.992; P <0.001).

Interestingly, as shown in Figure 5(b), the values of S_Crit1_ were equal to S_Crit2_ when both were computed from the same two distances, only (for example, 1.5 and 10 or 3 and 10 km). Similarly, ADC_1_ and ADC_2_ were equal when both were only computed from the same two distances.

For all the runners, the correlation coefficients for the linear regressions between 1/t_lim_ and D_lim_ in S_Crit2_ model were lower than for the t_lim_-D_lim_ regressions in S_Crit1_ model. In contrast, the ratios of estimated to actual speeds (Table 7) were more accurate in the S_Crit2_ model: the errors on 1500 m and RMSE were lower (P = 0.031) than in the S_Crit1_ model. On the other hand, the errors on 10000 m were higher (P = 0.031) in the S_Crit2_ model.

#### 4.2.4. Morton's Model Applied to Elite Runners

**Figure 6:**
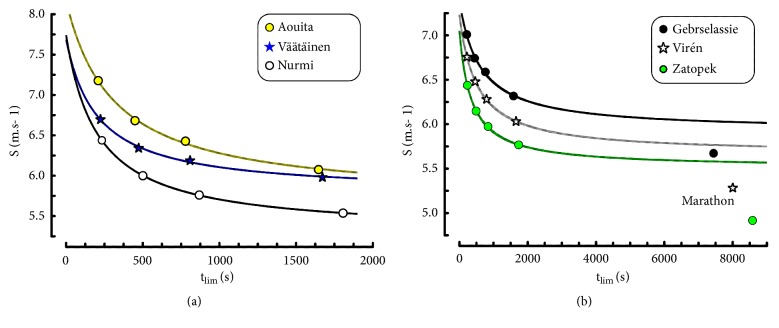
Relationship between running speed (S) and time (t_lim_) in Morton's model computed from 1500 to 10000 m. (b) The same model in the three subjects who ran the marathon.

In all the runners, the performances estimated from Morton's model were very close to their actual performances (Figure 6). When the 3-parameter model by Morton was computed with 4 distances (from 1500 m to 10000 m), the correlation coefficient was very high (0.999 ± 0.000752) in all the runners. When this model was computed with 3 distances (1500-3000-5000 m or 3000-5000-10000 m), the correlation coefficients were equal to 1 in all the runners.

The differences in S_Crit_, S_Max_, and ADC between the ranges of distances (Table 8) were all significant (P = 0.031). The ratios of estimated to actual speeds are presented in Table 9. In all the runners, the errors were very low (< 0.5%) for all the distances, from 1500 to 10000 m. However, the values of S corresponding to a marathon were overestimated in the three runners who participated in this road competition (Figure 6(b)).

### 4.3. Logarithmic Model Applied to Elite Runners

The values of parameters E and MAS in the logarithmic model depended on the range of running distance (Table 10) but these differences were not significant for MAS between 1500-10000 and 1500-5000 ranges and for E between 1500-5000 range and the two other distance ranges (P = 0.063).

The correlation coefficients were high, 0.995 ± 0.005, for the logarithmic model including the four distances from 1500 to 10000 m. The ratios of estimated to actual speeds for the four distances were accurate (Table 11): all the errors were lower than 1%.

When the 1500 m distance was not included as suggested by Péronnet and Thibault [7, 8], the correlation coefficient was higher (0.999 ± 0.002). The individual running performances between 3000 and 10000 m were well described by the logarithmic model as shown by the linear regressions between speed and the logarithm of t_lim_ (Figure 6(a)).

All the individual 1500m performances were above the individual regression lines computed from 3000 to 10000 m (Figure 6(a)) as in the logarithmic model including the 1500 m performances (Table 10).

**Figure 7:**
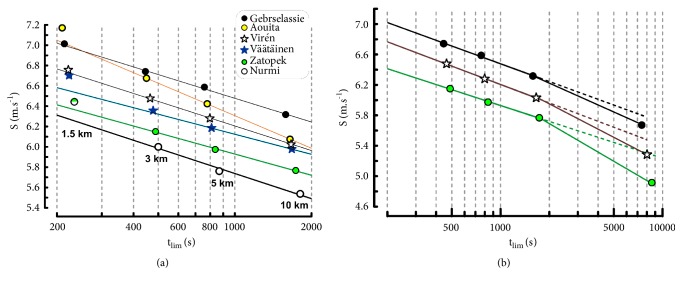
(a) Individual linear regressions between the logarithms of t_lim_ and running speeds. The data corresponding to 1.5 km were not included in the computation of the regressions. The performances by Nurmi and Zatopek were the same for the 1500 m distance. (b) Extrapolation of the speed-ln(t_lim_) relationships of the 3000-10000 m performances to t_lim_ corresponding to a marathon (dashed lines). The scale of t_lim_ is a logarithmic scale.

On the other hand, marathon performances were under the extrapolation of the lines of regression computed from the 3000-10000 m track performances (Figures 7(a) and 7(b)).

### 4.4. Exponential Models Applied to Elite Runners

**Figure 8:**
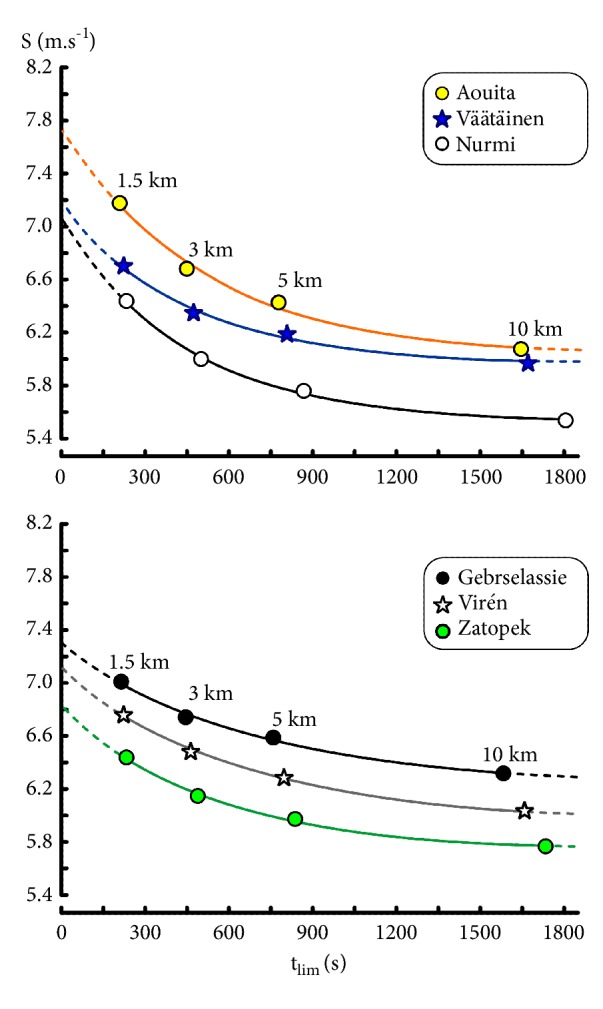
Individual relationships between running speed and t_lim_ in the Hopkins model computed with 4 distances (1500-10000 m).

The relationships between t_lim_ and S in the exponential model are presented in Figure 8.

As for the other models, the values of parameters S_∞_, S_0_, and 1/*τ* depended on the range of t_lim_-D_lim_ (Table 12).

When computed from 4 distances (Figure 8), the individual regressions were accurate (r = 0.998 ± 0.0014). Similarly, the ratios of estimated to actual speeds for the four distances were highly accurate (Table 13): all the errors were lower than 0.75%. As expected, the 3-parameter model was more accurate (r = 1) for the description of the elite runner performances when it was computed from 3 distances (1.5-3-5 km or 3-5-10 km), only.

### 4.5. Prediction of Running Speeds

#### 4.5.1. Prediction of Maximal Aerobic Speed

Maximal aerobic speed (MAS) can be estimated by computing the maximal speed corresponding to 7 min [7, 8] from the different models. These estimations (Table 14) were performed by interpolation from the 1500-5000m performances.

The effect sizes were small for all the differences (0.037 < Cohen's d < 0.218). The estimations of MAS were almost equal for S_Crit1_ and S_Crit2_ models that were significantly lower than the estimations of all the other models. The differences between all the other models were not significant (P ≥ 0.063).

The correlations between the different estimations were highly significant (r > 0.998; P < 0.001).

#### 4.5.2. Prediction of Maximal Speed during 30 Min

The estimations of the maximal running speed during 30 min done by extrapolation from the 1500-5000m performances are compared with the 10000 m performances (S_10000_) in Table 15. The correlations between the different estimations were highly significant (r ≥ 0.860; P < 0.0025). All the different estimations were significantly correlated with S_10000_ (r ≥ 0.989; P < 0.001). The effect sizes were small for the power-law and logarithmic models (Cohen's d = 0.131) or for the hyperbolic and exponential models (Cohen's d = 0.033) but large for the difference between power-law and exponential models (Cohen's d = 0.742). The 30-minute running speed estimated from asymptotic models was significantly higher than those estimated from power-law and logarithmic models (P = 0.031). The 30-min running speed was overestimated by the hyperbolic and exponential models because these estimations were approximately 2.5% higher than S_10000_ (P = 0.031) although the individual values of t_lim_ corresponding to 10000 m (Table 2) were lower than 1800 s (from 1583 to 1734 s) except for Nurmi (1806 s). On the contrary, the 30-minute estimated speeds computed with the logarithmic and power-law models were probably close to the actual 30-minute performances since they were slightly lower (0.7 and 1.4%) than S_10000_.

#### 4.5.3. Prediction of Maximal Speed during 60 Min

The estimations of maximal running speed during 60 min (Table 16) were done by extrapolation from the 1500-10000 m performances. The effect size between power-law and logarithmic models was small (Cohen's d = 0.073). All the predictions of the 60-min speeds from the different models were significantly correlated (r ≥ 0.964; P < 0.002). However, the 60-minute running speed predicted from the asymptotic models was significantly higher (P = 0.031) than those estimated from power-law and logarithmic models. Moreover, the prediction of the 60-minute running speed from the power-law model was higher than that from the logarithmic model (P = 0.031). It is possible that the 60-minute running speeds estimated from power-law and logarithmic models were slightly overestimated because the world record on one hour by Gebrselassie was about 2.5% slower (5.913 m.s^−1^ instead of 6.04 m.s^−1^ for the logarithmic model and 6.08 m.s^−1^ for the power-law model). On the other hand, the record by Zatopek on 20 km (3591 s; 5.57 m.s^−1^) was slightly faster than the 60-minute running speeds S estimated from the power-law (5.52 m.s^−1^) and logarithmic (5.50 m.s^−1^) models.

#### 4.5.4. Prediction of Marathon Performances

**Figure 9:**
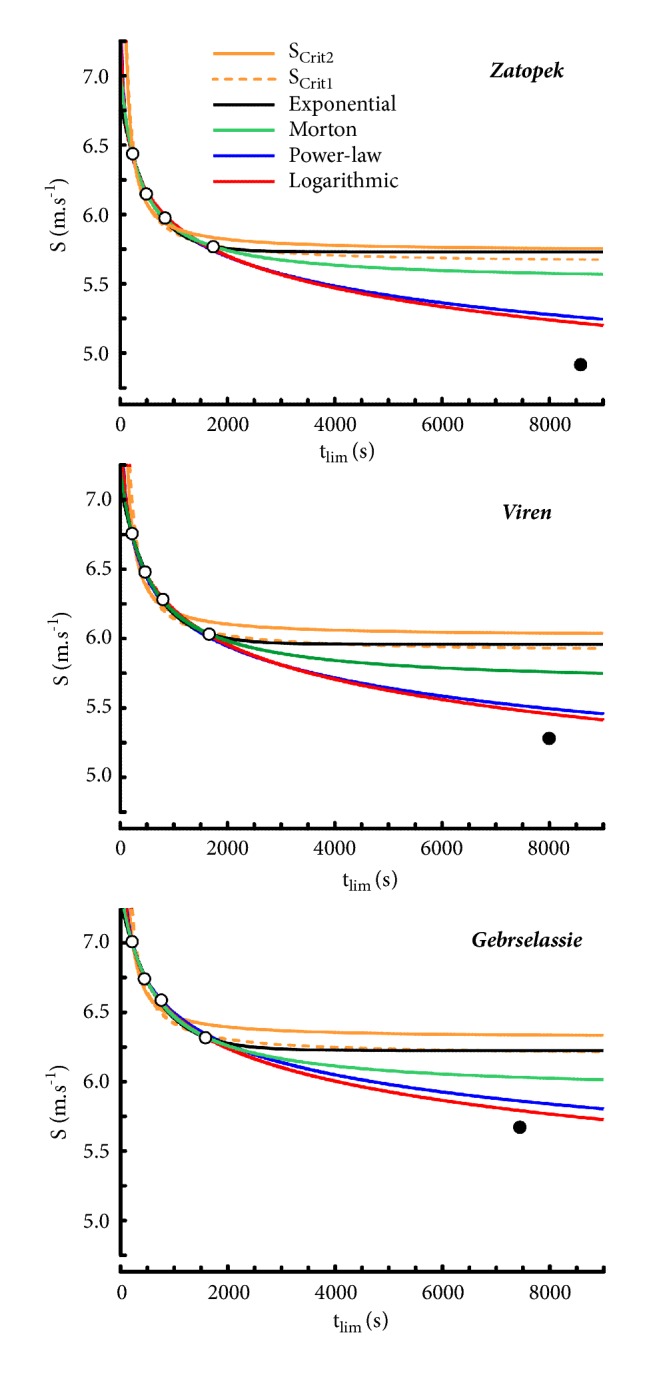
Comparisons of the relationship between (t_lim_) and running speed (S) of the logarithmic model, power-law model, S_Crit1_ and S_Crit2_ models, Morton's model, and exponential model computed from 4 distance performances (1500, 3000, 5000, and 10000 m; empty circles) in the three runners who participated in marathon (black dots).

The overestimations of the marathon running speed (Figure 9) by the different models were similar in the 3 runners. The predictions of marathon running speeds from the logarithmic model (red curves in Figure 9) were 5.216 m.s^−1^ for Zatopek, 5.457 m.s^−1^ for Viren, and 5.792 m.s^−1^ for Gebrselassié, which corresponded to overestimations equal to 6.1%, 3.4%, and 2.1%, respectively. The overestimations by the power-law model (blue curves in Figure 9) were slightly higher than those of the logarithmic model in the 3 runners.

On the other hand, the overestimations were more important with the four asymptotic models (hyperbolic models and exponential model). These overestimations by the asymptotic models were similar for the 3 runners who ran the marathon distance. The large overestimations were similar for the S_Crit1_ and S_Crit2_ models (orange curves) and exponential model (black curve). In the 3 marathon runners, the lowest overestimations by an asymptotic model corresponded to Morton's model (green curves).

### 4.6. Comparison of the Accuracies of the Different Models

For the modelling of the four distances (from 1500 to 10000 m), the lowest mean values of the RMSE of the six runners corresponded to Morton's model (Table 17).

The statistical significance values of the differences of the squared errors between the different models for the four distances and six runners (n = 24) are presented in Table 18. The accuracy of Morton's model was significantly better than those of all the other models. The accuracies of the power-law and logarithmic models were not statistically different. The accuracies of S_Crit1_ and S_Crit2_ models were not statistically different but were significantly lower than those of all the other models.

### 4.7. Correlations between the Parameters of the Different Models

#### 4.7.1. Correlations of the Endurance Indices

In Table 19, the comparisons of the endurance indices concern the indices computed with the running performances from 1500 to 5000 m that corresponded to the usual range of t_lim_ (3.5 to 15 min) in the studies on the modelling of the individual performances in nonelite runners. The correlations between the dimensionless indices (E and g) and either S_Crit1_ or S_Crit3_ or S_∞_ were not significant. In contrast, S_Crit1_, S_Crit3_, and S_∞_ were significantly correlated.

When S_Crit1_ was normalised to an estimate of maximal aerobic speed (S_420_) computed from the same model (Table 14), its correlations with the dimensionless indices g and E became significant (Table 19). After normalisation to S_420_ computed from the same model (Table 14), the correlation coefficients between S_Crit3_ or S_∞_ and the dimensionless indices (E and g) increased but were not significant.

#### 4.7.2. Correlations between S_Max_, S_0_, and k

When k, S_Max_, and S_0_ were computed from the performances in the 4 distances (from 1500 to 10000 m, Tables 2, 8, and 12), these parameters were significantly correlated (P ≤ 0.044):

(32) Smax=0.0617+1.040S0r=0.824k=−3.923+1.770Smaxr=0.862k=−7.55+2.357S0r=0.910

Parameter S_Max_ was significantly higher than S_0_ (P = 0.031). Parameter k was significantly higher than S_Max_ and S_0_ (P = 0.031).

When S_Max_, S_0_, and k were computed from 3 distance performances (1500-3000-5000) their values were significantly higher (P = 0.031) for S_Max_ and S_0_ but there was no significant correlation between S_Max_, S_0_, and k (r ≤ 0.788; P ≥ 0.063).

## 5. Discussion

Interestingly, for a given distance and a given model, the ratios of estimated to actual speeds were similar for the six runners (Tables 3, 5, 7, 9, 11, and 13). Indeed, for a given distance and a given model, the ratios of estimated to actual speed were not spread around 1 but either all the ratios were higher than 1 or all were lower (except several runners in the power-law model and one in the logarithmic model). Therefore, the modelling of the running performances was probably similar for the six elite runners although they ran in different conditions and they were probably trained according to different programmes. However, it cannot be excluded that there were submaximal performances in some runners. Indeed, the models would be similar if the ratios of submaximal speeds to maximal speeds are the same for each distance in a runner.

### 5.1. Effects of the Range of t_lim_

In the present study, there were significant differences in the parameters computed from the 3 different ranges of distances for the 3 hyperbolic models and the exponential model.

**Figure 10:**
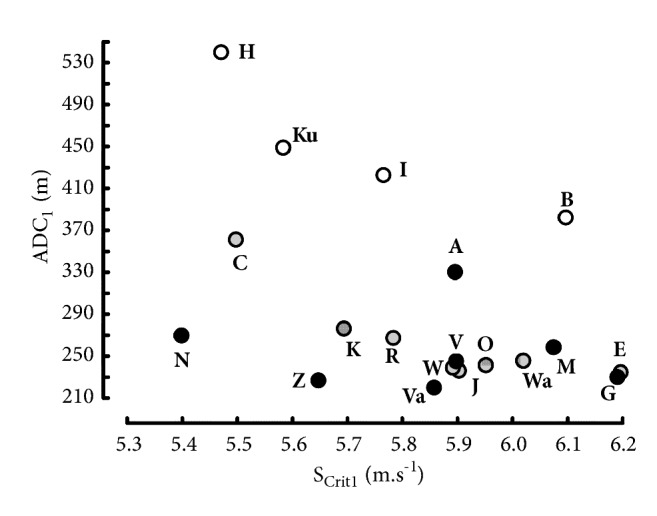
Relation between S_Crit1_ and ADC_1_ in the 19 elite runners whose ranges of performances were different: 1500-10000 m (black dots), 5000-10000 m (empty circles), and 3000-5000 m (grey dots).

The effect of the range of t_lim_ on a parameter is the most important for parameter ADC computed from the 3 different hyperbolic models (Figure 3 and Tables 4, 6, and 8). When the individual critical speeds decreased because of a change in the range of performances, the corresponding ADC increased. These increases in ADC_1_ (79%) were much larger than the decreases in S_crit1_ (3.8%) in the present study. The dependence of ADC on the range of performances can be verified (Figure 10) with the data of 19 elite endurance runners who were world-record holders and/or Olympic winners and/or world champions: Aouita (A), Bekele (B), Coe (C), El Gerrouj (E), Gebreselassie (G), Halberg (H), Ifter (I), Jazy (J), Keino (K), Kuts (Ku), Mo Farah (M), Nurmi (N), Ovett (O), Ryun (R), Väätäinen (Va), Viren (V), Wadoux (W), Walker (WA), and Zatopek (Z). The values of ADC_1_ were high (448 ± 67 m) in elite runners whose data included 5000 and 10000 m, only (empty circles). The values of ADC_1_ were lower (254 ± 38 m) in elite runners whose data included all the distances from 1500 to 10000 m (black dots). In elite runners whose data did not include the 10000 m performances, ADC_1_ were intermediate (263 ± 43 m). Moreover, the values of ADC are much higher in Morton's model (Table 8) than in S_Crit1_ and S_Crit2_ models (Tables 4 and 6). Therefore, the anaerobic capacity cannot be estimated from the hyperbolic models.

### 5.2. Endurance Indices

Parameter E of the logarithmic model by Péronnet and Thibault is an estimation of endurance capability [7, 8]. However, the validity of parameter E as an endurance index is questionable because MAS is computed assuming that the value of t_lim_ corresponding to MAS (t_MAS_) is equal to 7 min (420s) [7], which is contested. Indeed, in a review on the exhaustion time at V_O2_max [53], the value of t_MAS_ was 6 min. In another study on the energetics of the best performances in middle distance running [9] the value of t_MAS_ was estimated as equal to 14 min. Therefore, the interest of parameter E as an endurance index can be questioned because it depends on t_MAS_.

The effect of t_MAS_ on the endurance index by Péronnet-Thibault can be calculated [54]:

(33) S=α1  -  β1ln⁡tlim420MAS420=α1E420=100β1α1

If T = t_MAS_

(34) S=α2  -  β2ln⁡tlimTMAST=α2ET=100β2α2S=α1+β1ln⁡420-  β1ln⁡tlimS=α2+β2ln⁡T-  β2ln⁡tlim

The slopes between S and t_lim_ are the same. Therefore

(35) β1=β2S=α1+β1ln⁡420-  β1ln⁡tlim=α2+β1ln⁡T-  β1ln⁡tlimα1+β1ln⁡420=α2+β1ln⁡Tα2=α1+β1ln⁡420  -  β1ln⁡T=α1  -  β1ln⁡T420ET=100β2α2=100β1α1  -  β1ln⁡T/420ETE420=100β1/α1  -β1ln⁡T/420100β1/α1=α1α1  -  β1ln⁡T/420ETE420=11  -β1/α1ln⁡T/420=11  -  E420ln⁡T/420/100

**Figure 11:**
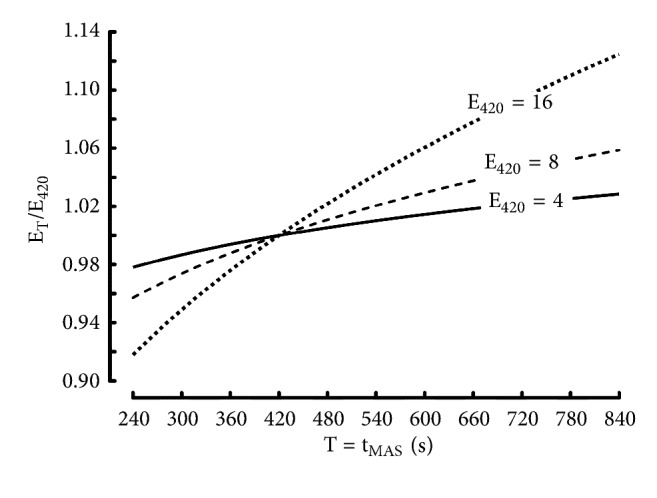
Effect of t_MAS_ (T) on the ratio E_T_/E_7min_ for an elite endurance runner (E_7min_ = 4), a medium level endurance runner (E_7min_ = 8), and a low-level endurance runner (E_7min_ = 16).

In Figure 11, this relationship between ratio E_T_/E_420_ and T (see (35)) is computed for 3 theoretical runners: an elite endurance runner (E_420_ = 4), a medium level endurance runner (E_420_ = 8), and a low level endurance runner (E_420_ = 16). The effect of t_MAS_ is much more important in the low level endurance runner than in the elite endurance runner (Figure 10).

Large variations in t_MAS_ have small effects on the classification of runners because the differences in E_420_ between elite and medium or low level runners are very large (from 4 to 16). For example, if t_MAS_ is equal to 14 min instead of 7 min, the medium level endurance runner would still be considered as a medium level endurance runner in spite of the increase of E (8.47 instead of 8). Similarly, the elite endurance runner would still be considered as an elite runner in spite of the increase in E (4.11 instead of 4) if t_MAS_ is also equal to 14 min instead of 7 min. On the other hand, if t_MAS_ is equal to 4 min instead of 7 min, the medium level endurance runner would still be considered as a medium level endurance runner in spite of the decrease in E (7.66 instead of 8.00). Similarly, the low level endurance runner would still be considered as a low level endurance runner in spite of the decrease in E (14.7 instead of 16) if t_MAS_ is also equal to 4 min instead of 7 min.

The endurance capability can also be estimated by the asymptotic models if parameters S_Crit1_, S_Crit2_, S_Crit3_, and S_∞_ are normalised to maximal aerobic speed (MAS). However, the values of MAS computed from the asymptotic models also depend on t_MAS_. Therefore, the validity of these endurance indices is questionable.

Parameter g of the power-law model by Kennelly has a high interest because it can be demonstrated that exponent g is a dimensionless index of endurance that does not depend on t_MAS_ unlike parameter E in the logarithmic model. The curvature of the D_lim_-t_lim_ equation depends on exponent g. In the elite endurance runners the D_lim_-t_lim_ equation is almost perfectly linear (Figure 2) whereas this equation is more curved in runners who are not endurance athletes. For example, exponent g was close to 1 in elite endurance runners and lower than 0.9 in physical education students [55]. It can be demonstrated that exponent g is equal to the ratio of the slope of the D_lim_-t_lim_ equation to MAS when t_lim_ is equal to t_MAS_. Indeed, the slope of D_lim_-t_lim_ is equal to the first derivative of the power-law equation. Therefore, the slope of the D_lim_–t_lim_ equation is equal to

(36) $$\begin{array}{c} \frac{{dD}_{lim}}{{dt}_{lim}}=\frac{d\left({{kt}_{lim}}^{g}\right)}{{dt}_{lim}}={{kgt}_{lim}}^{g\text{ – }1} \end{array}$$

For t_lim_ equal to t_MAS_, the running speed corresponds to MAS:

(37) S=MAS=ktMASg  -  1k=MAStMASg  –  1=MAStMAS1  –  g

Therefore

(38) $$\begin{array}{c} \frac{{dD}_{lim}}{{dt}_{lim}}=\left(\;{{MASt}_{MAS}}^{1\text{ – }g}\;\right)\left(\;{{gt}_{lim}}^{g\text{ – }1}\;\right) \end{array}$$

When t_lim_ = t_MAS_,

(39) dDlimdtMAS=MAStMAS1  –  ggtMASg  –  1=gMASdDlim/dtMASMAS=g

**Figure 12:**
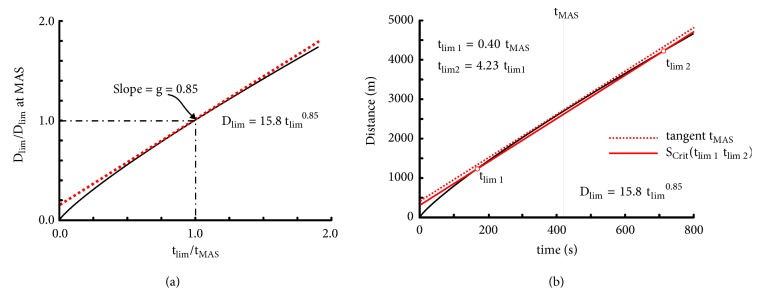
(a) Slope of the tangent at t_MAS_ of the curve corresponding to the power-law model with t_lim_ normalised to t_MAS_ and D_lim_ normalised to D_lim_ at maximal aerobic speed (MAS). (b) Comparison of a critical speed computed from two values of t_lim_ with the tangent at t_MAS_ (420s).

Consequently, the ratio of the D_lim_-t_lim_ slope to MAS corresponding to t_MAS_ is equal to exponent g and is independent of t_MAS_ unlike the endurance indices computed from the other models. In Figure 12(a), D_lim_ and t_lim_ are normalised to D_MAS_ (D_lim_ at MAS) and t_MAS_, respectively.

(40) DlimDMAS=DlimtMASMAS=ktlimgtMASMAS=MAS/tMASg  –  1tlimgtMASMAS=tlimtMASg

The slope of the line joining two points corresponding to t_lim1_ and t_lim2_ of the D_lim_-t_lim_ curve in Figure 12(b) is equal to exponent g when it is parallel to the tangent of the curve at t_MAS_. In Figure 12(b), ratio t_lim1_/t_mas_ is equal to 0.4 and ratio t_lim2_/t_lim1_ is equal to 4.23. In many studies on S_Crit_ (or P_Crit_) the range of t_lim_ is from 3 to 15 min, which corresponds to t_lim1_ equal to about 0.4-0.5 t_MAS_ (if t_MAS_ corresponds to 7 or 6 min) and ratio t_lim2_/t_lim1_ about 4-5. This range of t_lim_ also corresponds to the performances on 1500 and 5000 m in endurance runners. In the present study, when S_Crit1_ is computed from 1500-3000-5000m and is normalised to S_420_ (Table 14), the value of S_Crit1_/S_420_ is equal to 0.934 ± 0.016 and is significantly correlated (r = 0.976; P < 0.001) to g (0.934 ± 0.16). The product of exponent g and MAS is the equivalent of a critical speed computed from a 3-15-minute t_lim_ range. For example, the product of exponent g and S_420_ estimated from power-law model (Table 14) is equal to 6.04 ± 0.30 m.s^−1^ and is significantly correlated (r = 0.998; P < 0.001) with S_Crit1_ that is slightly but significantly (P = 0.031) lower (5.99 ± 0.31 m.s^−1^). The similar values of S_Crit_/S_420_ and g and the close values of S_Crit1_ and product g∗S_420_ and their significant correlation confirm the hypothesis that exponent g is an endurance index.

### 5.3. Correlations between the Parameters of the Different Models

The correlation between g and E was highly significant (r = 0.999, Table 19), which confirms the hypothesis that exponent g is an endurance index. Parameters S_Crit1_, S_Crit2_, S_Crit3_, and S_∞_ were highly correlated (P ≥ 0.965). These parameters that depend not only on endurance capability but also on maximal aerobic speed were not correlated with dimensionless parameters g and E (r ≤ 0.551). When S_Crit1_, S_Crit3_, and S_∞_ were normalised to an estimate of maximal aerobic speed (S_420_) computed from their model (Table 14), these parameters became dimensionless. The value of S_Crit1_/S_420_ was significantly correlated with the dimensionless indices g, and E (Table 19). After normalisation to S_420_, the correlation coefficients between S_Crit3_/S_420_ or S_∞_/S_420_ and E or g increased (r ≥ 0.676) but were not significant perhaps because of the small number of runners. Indeed, a correlation coefficient equal to 0.6664 would have been significant if there were 9 runners.

A study [56] compared the critical speeds from different mathematical models in 12 middle- or long-distance male runners on a track in order to determine which model provides the most accurate prediction of performance in 1 hour. In this latter study, the parameters S_Crit1_, S_Crit2_, S_Crit3_, and S_∞_ were also significantly correlated (0.85 < r < 0.99, p < 0.01) and the differences between these different critical speeds were the same as in the present study for the 1500-5000 m range: S_Crit3_ < S_Crit1_ < S_Crit2_ < S_∞_.

The meaning of parameters S_Max_ (Morton's Model) and S_0_ (exponential model) is identical and corresponds, in theory, to maximum running speed. When S_Max_ and S_0_ were computed from the 4 distance performances (from 1500 to10000 m, Tables 8 and 12), these parameters were significantly correlated (r = 0.824; P = 0.044). However, S_Max_ was significantly higher than S_0_ (P = 0.31). When S_Max_ and S_0_ were computed from the 3 distance performances (from 1500 to 5000 m) their values were higher. A previous study [57] compared which parameter (S_Max_ or S_0_) is closest to maximum speed by measuring maximal velocity during a sprint. The values of S_Max_ and S_0_ were well correlated (r = 0.93, P<0.001) but they were significantly different. As in the present study, S_Max_ (7.80 ± 0.93 m.s^−1^) was higher than S_0_ (7.49 0.90 m.s^−1^) but lower than the actual maximum speed (8.43 ± 0.33 m.s^−1^) on a track. However, S_Max_ and S_0_ were computed from the performances on a treadmill whereas the actual maximum running speed was measured on a track during short sprints with photocells placed at 30 and 40 m. It is likely that it would be better to measure actual maximum speed during a 60 m sprint on a track with a laser apparatus and to compare it with S_Max_ and S_0_ from Morton's model and exponential models computed from performances on a track instead of a treadmill.

In the present study, parameter k of the power-law model was 25% higher than S_Max_ and 31% higher than S_0_. However, k was significantly correlated with S_Max_ and S_0_. These results confirm the hypothesis that parameter k should be correlated with the maximal running speed because it is equal to the running speed corresponding to one second. However, the value of k depends on the time unit. If the running performances are evaluated in minutes, parameter k would be equal to the maximal speed corresponding to 1 minute whereas S_Max_ and S_0_ would still correspond to maximal running speed but expressed in m.min^−1^.

### 5.4. Prediction of Long Distances

The asymptotes of hyperbolic and exponential model correspond to S_Crit1_, S_Crit2_, S_Crit3_, and S_∞_, respectively. In these models, the speeds lower than these asymptotes can be maintained infinitely. Therefore, the extrapolations of the asymptotic hyperbolic and exponential models overestimate the running speeds on very long distances (Figure 9). In fact, power-law and logarithmic models are also asymptotic models but these asymptotes are equal to zero.

The overestimations of marathon performances from the extrapolations of power-law and logarithmic models (Figures 1(b), 6(b), and 9) are much smaller. Similarly, the computations of 30-minute and 60-minute running speeds by extrapolation of the asymptotic models (Table 7) were probably overestimations whereas the extrapolations of the power-law and logarithmic models were probably close to the actual running speeds.

The overestimations of marathon performances by the logarithmic and power-law models (Figures 1, 6, and 9) are probably due not only to the causes of fatigue in long distances [58] but, perhaps, also to the effects of ground (track versus road, slopes, etc.), wind, shoes, and age.

### 5.5. Which Is the Optimal Empirical Model?

The optimal running model is an accurate, useful, and practical model.

#### 5.5.1. Which Is the Most Accurate Model?

When computed from 4 distances, the individual correlation coefficients of all the models were high in all the elite runners. The correlation coefficients were the highest for the 3-parameter models by Morton and Hopkins and they were equal to 1 when they were computed from 3 distances only. These correlation coefficients equal to 1 were expected. Similarly, the regression coefficients of all the 2-parameter running models would have been equal to 1, if they were computed with only two distances.

The values of RMSE were the lowest for the 3-parameter models (Table 17). Morton's model was the most accurate as demonstrated by the ratios of estimated to actual running speeds which were very close to 1 for each distance (Table 9). Indeed, the differences between the estimated to actual running speeds were lower than 0.5% in each distance for all the runners. This model was significantly more accurate than all the other models as shown in Table 18.

However, if a running model is perfect, there should be no significant difference between its parameters computed from different ranges of distances. Morton's model was probably not perfect because its parameters were significantly different (P = 0.031) when they were computed from different ranges of distances. In the present study, the empirical models consist of single equations and are less complex than the physiological and biomechanical models, which probably explained that the parameters of all these empirical models depended on the range of t_lim_. Indeed, the causes of fatigue differ for short, medium, and long distances [58].

The S_Crit1_ and S_Crit2_ models and the concepts of critical speed (or critical power) are by far the most used and taught [21, 46]. Nonetheless, S_Crit1_ and S_Crit2_ models were the less accurate models for the relationship between running speed and t_lim_. The curves derived from (12) and (14) did not describe accurately the relationships between speed and t_lim_ (Figures 4(b) and 4(c)). The only points corresponding to 10000 m performances were close to the curves derived from (12) whereas the only points corresponding to 1500 m performances were close to the curves derived from (14). Consequently, the speed-t_lim_ relationship would be better described by the mean values of ADC and S_Crit_:

(41) $$\begin{array}{c} ADC=\frac{\left({ADC}_{1}+{ADC}_{2}\right)}{2}=\frac{\left(\alpha_{1}+\beta_{2}\right)}{2}\\ S_{Crit}=\frac{\left(S_{Crit1}+S_{Crit2}\right)}{2}=\frac{\left(\alpha_{2}+\beta_{1}\right)}{2} \end{array}$$

**Figure 13:**
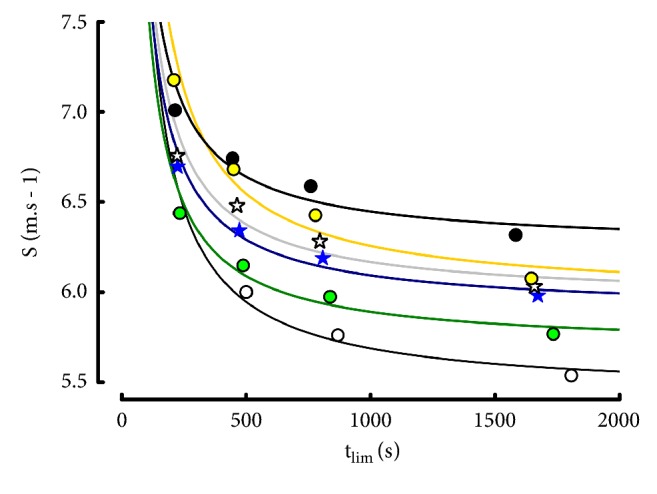
Individual relationships between speed and t_lim_ computed from the mean values of ADC and S_Crit_ in (12) and (14).

Even if the description of the individual speed-t_lim_ relationships was better with the curves computed from the mean values of ADC and S_Crit_ in (12) and (14) (Figure 13), this new hyperbolic model is not optimal when it is compared with the figures of the other models.

#### 5.5.2. Which Is the Most Useful Model?

The empirical models of running exercises are often used to estimate the running speeds over given distances, the endurance capability, and MAS. The race performance calculation requires 2 or 3 parameters depending on the model used. On the other hand, for each running model in the present study, there is only one parameter that is an expression of the long-distance running capability. Indeed, parameter ADC in the hyperbolic models is not reliable and parameters k, S_Max_, and S_0_ that are maximal speed indices are probably not useful for endurance runners. Similarly the parameter corresponding to the time constant (*τ*) in Hopkins' model is not useful.

The useful parameters of the asymptotic model correspond to S_Crit1_, S_Crit2_, S_Crit3_, and S_∞_. In theory, these parameters represent the fastest speed that can be maintained for a very long time. However, when S_Crit1_ was computed from exercises shorter than 20 min, the subjects were generally only able to maintain S_Crit1_ for less than 30 min and the running velocities that could be maintained for 60 minutes on a treadmill were largely overestimated by S_Crit1_ [59]. In another study on the relationship between critical velocity and marathon performance [60], S_Crit1_ (4.43 m.s^−1^) was 44% faster than the marathon running speed (3.07 m.s^−1^). Nonetheless, the correlation between marathon performance and S_Crit1_ was more significant than the correlations with the other physiological parameters. In this latter study, it was possible to calculate an approximation of the marathon performance from S_Crit1_ (r = 0.87 and SEE = 14 min). Approximations of long-distance performances (> 10000 m) are probably also possible with S_Crit2_, S_Crit3_, and S_∞_ since they are highly correlated with S_Crit1_ (P ≥ 0.965). For example, in the study on 12 trained middle- and long-distance male runners [56], the correlation coefficients of S_Crit1_, S_Crit2_, and S_∞_ with the maximal running speed during 60 min were equal to 0.90, 0.91, and 0.93, respectively. Amazingly, the correlation coefficient with the 60-min running speed was the lowest (0.80) for S_Crit3_ in these middle- and long-distance runners but the overestimation was the smallest (0.13 ± 0.21 m.s^−1^) as in the present study.

It is likely that the logarithmic and power-law models that are not asymptotic are the best empirical models for the predictions of very long distances by extrapolation as suggested in Table 15 and Figure 9. The predictions of the running speeds corresponding to 30 min, 60 min, and marathon by extrapolation of Morton's model were higher than the same predictions from the logarithmic and power-law models. But the overestimations of the running speeds corresponding to 30 min, 60 min, and marathon by Morton's model were lower than the overestimations by the other asymptotic models (Tables 15 and 16 and Figure 9). On the other hand, the predictions of competition performances between 1500 and 10000 m (for example, one or two miles or 2000 m) by interpolation should be better with the 3-parameter models by Morton or Hopkins whose accuracies were the best. Similarly, the running speed corresponding to 6 or 7 min (an estimation of MAS) should be more accurate when computed with these 3-parameter models.

The endurance index of the power-law model (exponent g) should be the most useful since it is the only endurance index that does not depend on t_MAS_ (Section 5.2).

#### 5.5.3. Which Is the Most Practical?

The most practical model should be the less sensitive to a slightly submaximal performance and the easiest to compute.

Unfortunately, no study compares the sensitivity of the different models to submaximal performances. However, in a previous study [61], some results were assumed to be the effect of submaximal performances on S_Crit1_ model whose sensitivity was discussed in a review on the critical power concept [16]. Similarly, the values of parameter k that is an index of maximal running speed were overestimated in several physical education students in a previous study [55], which was probably the effect of submaximal running performances. Indeed, in 4 physical education students, parameters k were largely overestimated since they were higher than 20 m.s^−1^, whereas the maximal running speed is about 12.2 m.s^−1^ for the best world sprinter U. Bolt [62]. The comparison of parameters k of Ovett and Coe [63] is also a demonstration of the effects of submaximal performances on the modelling of running performances with the power-law model. Indeed, the differences between Ovett and Coe for the performances over 800, 1500, and 2000 m are around 1 second but the inclusion of longer distances (3000 m and 5000 m) causes large differences in the values of k and g. The value of k was largely higher than 12 m.s^−1^ for Coe but not for Ovett. The best performance for a given distance is probably maximal if the elite runner has run this distance many times, which was not the case for Coe in the 3000 m and 5000 m distances. In the present study, the sensitivity of Morton's model to submaximal performances could be not negligible. Indeed, the parameters of this model were significantly different when they were computed from different distance ranges although the differences between the estimated and the actual speeds were very low (< 0.5%). The sensitivity of Morton's model to submaximal performances could also explain why the correlation coefficient of S_Crit3_ with the 60 min speed was the lowest in the study on the twelve middle- and long-distance runners [56].

Many runners compete over two distances, only (either 800 and 1500 m or 5000 and 10000 m or half-marathon and marathon). Their performances on the other distances could be slightly submaximal and, consequently, the 3-parameter models by Morton or Hopkins could be not optimal for these runners.

The 3-parameter models need a software that can compute the parameters by iteration. The 2-parameter models are easier to compute either by a nomogram [48] or by the current database software (Microsoft Excel, LibreOffice Calc, etc.). The calculation of S_Crit1_ is much easier than the parameters of the other models. Particularly, it is very easy to calculate S_Crit1_ from two running performances:

(42) $$\begin{array}{c} S_{Crit1}=\frac{\left(D_{lim2}\;\;–\;\;D_{lim1}\right)}{\left(t_{lim2}\;\;–\;\;t_{lim1}\right)} \end{array}$$

In addition, the S_Crit1_ model is the only model that can directly predict the performance corresponding to a distance from its parameters (ADC_1_ and S_Crit1_):

(43) $$\begin{array}{c} D_{lim}={ADC}_{1}+S_{Crit1}*t_{lim}\\ t_{lim}=\frac{\left(D_{lim}\text{ - }{ADC}_{1}\right)}{S_{Crit1}} \end{array}$$

In the present study, the other models can only predict performances corresponding to a value of t_lim_. In these models, the protocol presented in Section 3.3 is necessary for the prediction of a performance corresponding to a distance.

## 6. Conclusion

The comparison of the accuracies of the different models in the six elite endurance runners suggests that the most accurate model is the asymptotic 3-parameter hyperbolic model proposed by Morton and that the less accurate models are S_Crit1_ and S_Crit2_ models which are the most often used. However, it is likely that logarithmic and power-law models are the most accurate models for the predictions of long-distance performances (maximal running speeds for 30 and 60 min or marathon) by extrapolation. In addition, exponent g of the power-law model is an interesting endurance index that does not depend on t_MAS_. The comparison of the sensitivity of the different models to submaximal performances should be studied to select the most practical model.

## Figures and Tables

**Table 1:** Individual performances (in seconds) of elite endurance runners.

	*1500*	*3000*	*5000*	*10000*	*Marathon*
Nurmi	233	500	868	1806	
Zatopek	233	488	837	1734	8583
Väätäinen	224	473	808	1672	
Virén	222	463	796	1658	7991
Aouita	209	449	778	1646	
Gebrselassie	214	445	759	1583	7439

**Table 2:** Parameters k and g according to the ranges of distances used in the computation of the power-law model.

	1500-10000 m		1500-5000 m		3000-10000 m	
	k	g	k	g	k	g
Nurmi	9.55	0.926	10.2	0.915	8.80	0.938
Zatopek	8.65	0.945	8.86	0.941	8.39	0.950
Väätäinen	8.99	0.944	9.36	0.938	8.45	0.953
Virén	9.17	0.943	9.20	0.943	9.14	0.944
Aouita	11.0	0.920	11.3	0.915	10.5	0.927
Gebrselassie	9.24	0.949	9.12	0.951	9.25	0.948

**Mean**	**9.43**	**0.938**	**9.67**	**0.934**	**9.08**	**0.943**
**SD**	**0.81**	**0.012**	**0.90**	**0.015**	**0.76**	**0.010**

**Table 3:** Ratios of estimated to actual speeds for the different distances in the power-law model. RMSE = root mean square of the errors between estimated running speed and actual speed.

	1500	3000	5000	10000	RMSE
Nurmi	0.9909	1.0058	1.0057	0.9899	0.00794
Zatopek	0.9958	1.0016	1.0005	0.9954	0.00323
Väätäinen	0.9919	1.0051	0.9994	0.9924	0.00612
Viren	0.9974	0.9974	0.9975	0.9963	0.00290
Aouita	0.9965	1.0077	1.0022	0.9981	0.00448
Gebrselassié	1.0022	1.0039	0.9995	1.0042	0.00309

**Mean **	**0.996 **	**1.004**	**1.001**	**0.996 **	**0.00463**
**SD**	**0.0041**	**0.0037**	**0.0029**	**0.00495**	**0.00203**

**Table 4:** Values of S_Crit1_ and ADC of the S_Crit1_ model according to the range of distances. *∗*: P = 0.031 for all the differences between the different ranges.

	1500-10000 m		1500-5000 m		3000-10000 m	
	S_Crit1_	ADC_1_	S_Crit1_	ADC_1_	S_Crit1_	ADC_1_
Nurmi	5.39	284	5.51	228	5.35	339
Zatopek	5.65	226	5.79	160	5.61	282
Väätäinen	5.86	220	5.99	161	5.83	262
Virén	5.90	245	6.09	160	5.85	314
Aouita	5.89	332	6.14	225	5.83	413
Gebrselassie	6.19	230	6.42	133	6.13	301

**Mean**	**5.81** **∗**	**256** **∗**	**5.99** **∗**	**178** **∗**	**5.77** **∗**	**319** **∗**
**SD**	**0.27**	**44**	**0.31**	**39**	**0.26**	**53**

**Table 5:** Ratios of estimated to actual speeds for the different distances in the S_Crit1_ model. RMSE = root mean square of the errors between estimated running speeds and actual speeds.

	1500	3000	5000	10000	RMSE
Nurmi	1.032	0.992	0.992	1.002	0.0173
Zatopek	1.033	0.994	0.990	1.002	0.0175
Väätäinen	1.026	0.997	0.991	1.002	0.0138
Vir10n	1.044	0.992	0.988	1.003	0.0231
Aouita	1.056	0.993	0.983	1.004	0.0296
Gebrselassié	1.043	0.995	0.985	1.003	0.0231

**Mean **	**1.039 **	**0.994**	**0.988 **	**1.003**	**0.021**
**SD**	**0.011**	**0.002**	**0.004**	**0.001**	**0.006**

**Table 6:** Values of S_Crit2_ and ADC_2_ according to the range of distances. ∗: P = 0.031 for all the differences between the different ranges.

	1500-10000 m		1500-5000 m		3000-10000 m	
	S_Crit2_	ADC_2_	S_Crit2_	ADC_2_	S_Crit2_	ADC_2_
Nurmi	5.47	233	5.54	211	5.37	318
Zatopek	5.74	171	5.82	146	5.64	257
Väätäinen	5.93	176	6.00	156	5.86	235
Virén	6.02	174	6.14	141	5.88	283
Aouita	6.04	248	6.18	210	5.89	368
Gebrselassie	6.32	157	6.44	123	6.19	257

**Mean**	**5.92** **∗**	**193** **∗**	**6.02** **∗**	**165** **∗**	**5.81** **∗**	**286** **∗**
**SD**	**0.29**	**38**	**0.31**	**37**	**0.28**	**49**

**Table 7:** Ratios of estimated to actual speeds for the different distances in the S_Crit2_ model. RMSE = root mean square of the errors between estimated running speed and actual speed.

	1500	3000	5000	10000	RMSE
Nurmi	1.005	0.989	0.996	1.011	0.00834
Zatopek	1.005	0.990	0.994	1.012	0.00855
Väätäinen	1.003	0.994	0.994	1.009	0.00659
Viren	1.006	0.987	0.993	1.015	0.0112
Aouita	1.007	0.986	0.989	1.019	0.0132
Gebrselassié	1.006	0.989	0.990	1.016	0.0110

**Mean **	**1.005**	**0.989**	**0.993**	**1.014**	**0.00981**
**SD**	**0.0013**	**0.0027**	**0.0025**	**0.0035**	**0.00241**

**Table 8:** Values of S_Crit3_, S_Max_ and ADC of Morton's model according to the range of distances. ∗: P = 0.031 for all the differences between the different ranges.

	1500-10000 m			1500-5000 m			3000-10000 m		
	S_Crit3_	S_Max_	ADC	S_Crit3_	S_Max_	ADC	S_Crit3_	S_Max_	ADC
Nurmi	5.29	7.74	504	5.31	7.85	470	5.27	7.45	549
Zatopek	5.51	7.05	539	5.61	7.25	388	5.44	6.74	760
Vaatainen	5.78	7.69	393	5.94	9.70	211	5.59	6.77	982
Viren	5.67	7.23	793	5.77	7.31	605	5.60	7.08	995
Aouita	5.69	8.16	772	5.98	9.07	410	5.41	7.34	1666
Gebrselassié	5.92	7.40	868	6.28	7.85	292	5.46	7.05	2961

**Mean**	**5.64** **∗**	**7.55** **∗**	**645** **∗**	**5.82** **∗**	**8.17** **∗**	**396** **∗**	**5.46** **∗**	**7.07** **∗**	**1319** **∗**
**SD**	**0.22**	**0.40**	**193**	**0.33**	**0.99**	**137**	**0.12**	**0.29**	**888**

**Table 9:** Ratios of estimated to actual speeds for the different distances in Morton's model. RMSE = root mean square of the errors between estimated running speed and actual speed.

	1500	3000	5000	10000	RMSE
Nurmi	1.0000	1.0004	0.9995	1.0003	0.00036
Zatopek	0.9997	1.0012	0.9985	1.0005	0.00100
Väätäinen	0,9996	1.0027	0.9965	1.0014	0.00236
Viren	0.9997	1.0008	0.9990	1.0003	0.00069
Aouita	0.9993	1.0038	0.9953	1.0017	0.00315
Gebrselassié	0.9992	1.0033	0.9968	1.0010	0.00241

**Mean**	**0.9996**	**1.0020**	**0.9976**	**1.0009**	**0.0017**
**SD**	**0.0003**	**0.0014**	**0.0016**	**0.0006**	**0.0011**

**Table 10:** Values of MAS and E in the logarithmic model according to the range of distances. ^a^: P = 0.031 between 1500-10000 and 3000-10000 m;  ^b^: P = 0.031 between 1500-5000 and 3000-10000 m.

	1500-10000 m		1500-5000 m		3000-10000 m	
	MAS	E	MAS	E	MAS	E
Nurmi	6.13	7.18	6.12	8.48	6.05	5.90
Zatopek	6.22	5.35	6.22	5.87	6.19	4.83
Väätäinen	6.44	5.47	6.43	6.24	6.38	4.49
Virén	6.52	5.54	6.52	5.72	6.51	5.39
Aouita	6.77	7.82	6.76	8.52	6.71	6.96
Gebrselassie	6.78	5.05	6.78	4.94	6.77	4,98

**Mean**	**6.48**	**6.07**	**6.47**	**6.63**	**6.43** ^**a,b**^	**5.42** ^**a**^
**SD**	**0.27**	**1.14**	**0.27**	**1.51**	**0.29**	**0.90**

**Table 11:** Ratios of estimated to actual speeds for the different distances in the logarithmic model. RMSE = root mean square of the errors between estimated running speeds and actual speeds.

	1500	3000	5000	10000	RMSE
Nurmi	0.992	1.009	1.009	0.990	0.00916
Zatopek	0.997	1.004	1.003	0.996	0.00352
Väätäinen	0.993	1.008	1.002	0.994	0.00622
Viren	0.999	1.000	1.001	0.998	0.00135
Aouita	0.994	1.008	1.003	0.995	0.00594
Gebrselassié	0.999	1.002	0.998	1.001	0.00167

**Mean **	**0.996 **	**1.005**	**1.003 **	**0.996**	**0.00460**
**SD**	**0.0031**	**0.0037**	**0.0038**	**0.0037**	**0.00302**

**Table 12:** Values of S_∞_, S_0_ and 1/*τ* of the exponential model according to the range of distances. ∗: P = 0.031 for all the differences between the different ranges.

	1500-10000 m			1500-5000 m			3000-10000 m		
	S_∞_	S_0_	1/*τ*	S_∞_	S_0_	1/*τ*	S_∞_	S_0_	1/*τ*
Nurmi	5.52	7.06	0.00224	5.64	7.24	0.00298	5.48	6.68	0.00167
Zatopek	5.73	6.81	0.00187	5.87	6.96	0.00280	5.68	6.57	0.00132
Vaatainen	5.97	7.17	0.00228	6.13	7.50	0.00397	5.86	6.69	0.00115
Virén	5.96	7.10	0.00163	6.11	7.20	0.00234	5.91	6.94	0.00127
Aouita	6.03	7.76	0.00202	6.31	8.13	0.00354	5.87	7.23	0.00114
Gebreselassie	6.23	7.29	0.00151	6.50	7.52	0.00323	5.99	7.03	0.00073

**Means**	**5.91** **∗**	**7.20** **∗**	**0.00193** **∗**	**6.09** **∗**	**7.43** **∗**	**0.00314** **∗**	**5.80** **∗**	**6.86** **∗**	**0.00121** **∗**
**SD**	**0.25**	**0.32**	**0.00032**	**0.31**	**0.40**	**0.00057**	**0.19**	**0.25**	**0.00031**

**Table 13:** Ratios of the estimated to actual speeds in the different distances for the exponential model. RMSE = root mean square of the errors between estimated running speeds and actual speeds.

	1500	3000	5000	10000	RMSE
Nurmi	0.999	1.004	0.996	1.002	0.00293
Zatopek	0.999	1.003	0.997	1.001	0.00227
Väätäinen	0.998	1.005	0.994	1.002	0.00405
Viren	0.999	1.002	0.998	1.001	0.00162
Aouita	0.998	1.007	0.993	1.002	0.00530
Gebrselassié	0.998	1.005	0.996	1.001	0.00309

**Mean **	**0.999**	**1.004**	**0.996 **	**1.001 **	**0.00321 **
**SD**	**0.0005**	**0.0018**	**0.0018**	**0.0006**	**0.00131**

**Table 14:** Estimation of maximal running speed (m.s^− 1^) corresponding to 420 s computed from the different models. ∗: P = 0.031 for the differences with Morton's model, exponential, logarithmic and power-law models.

	*SCrit1*	*SCrit2*	*Morton*	*Exponential*	*Log*	*Power*
Nurmi	6.0491	6.0454	6.09	6.10	6.12	6.11
Zatopek	6.1714	6.1669	6.20	6.21	6.22	6.20
Väätäinen	6.3716	6.3737	6.39	6.39	6.43	6.41
Virén	6.4693	6.4690	6.52	6.52	6.52	6.53
Aouita	6.6776	6.6812	6.72	6.72	6.76	6.75
Gebrselassie	6.7348	6.7349	6.76	6.76	6.78	6.79

**Means**	**6.412** **∗**	**6.412** **∗**	**6.45**	**6.45**	**6.47**	**6.47**
**SD**	**0.272**	**0.274**	**0.27**	**0.27**	**0.27**	**0.28**

**Table 15:** Maximal running speed (m.s^−1^) during 30 min, computed from the different models. S_10000_: running speed over 10000 m; ∗: P = 0.031 for the differences with logarithmic and power-law models. ^1^: P = 0.031 for the differences with S_Crit1_ model. ^3^: P = 0.031 for the differences with Morton's model. ^S^: P = 0.031 for the differences with S_10000_.

	Log	Power	Morton	S_Crit1_	Exp	S_Crit2_	S10000
Nurmi	5.36	5.40	5.55	5.63	5.65	5.66	5.54
Zatopek	5.69	5.69	5.80	5.88	5.88	5.90	5.77
Väätäinen	5.84	5.85	6.06	6.08	6.13	6.09	5.98
Virén	5.98	6.01	6.05	6.18	6.13	6.21	6.03
Aouita	5.92	5.96	6.19	6.27	6.31	6.30	6.07
Gebrselassie	6.29	6.32	6.43	6.49	6.50	6.52	6.32

**Mean**	**5.85** ^S^	**5.87**	**6.01** **∗**,^S^	**6.09**∗^, 3, S^	**6.10**∗^,3, S^	**6.11**∗^,1,3,S^	**5.95**
**SD**	**0.31**	**0.31**	**0.30**	**0.30**	**0.31**	**0.30**	**0.27**

**Table 16:** Maximal running speed (m.s^−1^) during 60 min computed from the different models. ∗: P = 0.031 for the differences with logarithmic model. ^P^: P = 0.031 for the differences with power-law model. ^1^: P = 0.031 for the differences with S_Crit1_ model. ^3^: P = 0.031 for the differences with Morton's model. ^E^: P = 0.031 for the differences with exponential model.

	Log	Power	Morton	S_Crit1_	Exp	S_Crit2_
Nurmi	5.18	5.21	5.42	5.47	5.52	5.53
Zatopek	5.50	5.52	5.65	5.71	5.73	5.78
Väätäinen	5.68	5.69	5.88	5.92	5.96	5.98
Virén	5.74	5.75	5.86	5.97	5.95	6.07
Aouita	5.63	5.69	5.89	5.99	6.02	6.11
Gebrselassie	6.04	6.08	6.13	6.25	6.22	6.36

**Mean**	**5.63**	**5.66** **∗**	**5.81** **∗** ^,P^	**5.88** **∗** ^,P,3^	**5.91** **∗** ^,P,3^	**5.97** **∗** ^,P,1,3,E^
**SD**	**0.28**	**0.29**	**0.24**	**0.27**	**0.25**	**0.29**

**Table 17:** Average values of the 6 runners Roots Mean Square Errors (RMSE) for the different models.

	RMSE
Morton's model	0.00166 ± 0.00113
Exponential model	0.00321 ± 0.00131
Power-law model	0.00463 ± 0.00203
Logarithmic model	0.00464 ± 0.00302
S_Crit2_ model	0.00981 ± 0.00241
S_Crit1_ model	0.0207 ± 0.00566

**Table 18:** Values of paired Student't test (underlined) or Wilcoxon signed rank test for the difference in squared errors between the running models.

	Power law	S_Crit1_	S_Crit2_	Morton	Logarithmic	Exponential
Power law	X					

S_Crit1_	0.003	X				

S_Crit2_	< 0.001	0.484	X			

Morton	0.001	0.001	< 0.001	X		

Logarithmic	0.830	< 0.001	< 0.001	0.005	X	

Exponential	0.061	< 0.001	< 0.001	< 0.001	0.017	X

**Table 19:** Correlation coefficients of the linear regressions between the different endurance indices. ∗: P = 0.05; ∗∗∗: P <0.001.

	S_Crit1_	S_Crit3_	g	E	S_Crit1_/S_420_	S_Crit3_/S_420_
S_Crit1_	X					

S_Crit3_	0.965∗∗	X				

g	0.551	0.513	X			

E	0.538	0.499	0.999∗∗∗	X		

S_∞_	0.985∗∗∗	0.984∗∗∗	0.435	0.422		

S_Crit1_/S_420_			0.976∗∗∗	0.973∗∗∗	X	

S_Crit3_/S_420_			0.683	0.676	0.775	X

S_∞_/S_420_			0.720	0.711	0.824∗	0.991∗∗∗

## Data Availability

All the “experimental” data are presented in Table 1. All the results of the computations according to the different models are presented in the next 15 tables (from Table 2 to Table 16).
